# Refractory Takayasu arteritis responding to the oral Janus kinase inhibitor, tofacitinib

**DOI:** 10.1093/rap/rkz050

**Published:** 2019-12-23

**Authors:** Yuriko Yamamura, Yoshinori Matsumoto, Yosuke Asano, Yu Katayama, Keigo Hayashi, Keiji Ohashi, Michiko Morishita, Haruki Watanabe, Mariko Takano-Narazaki, Ken-Ei Sada, Jun Wada

**Affiliations:** Department of Nephrology, Rheumatology, Endocrinology and Metabolism, Okayama University Graduate School of Medicine, Dentistry and Pharmaceutical Sciences, Okayama, Japan

Sir, Takayasu arteritis (TAK) is a rare autoinflammatory disease, characterized by aortitis affecting the aorta and its major branches, coronary arteries and pulmonary arteries [1]. Long-term inflammation often causes severe vascular injury, including thickening of the aorta and its main branches, fibrosis, stenosis and thrombus formation, leading to organ failure and subsequent numerous symptoms attributable to ischaemia [1, 2]. The pathogenesis of TAK is yet to be elucidated, leading to limitations in therapeutic strategies and prognosis.

Tofacitinib (TOF) is an oral Janus kinase inhibitor, which blocks cytokine-mediated inflammatory signalling through the suppression of Janus kinase 1 and 3 [3]. Tofacitinib is clinically indicated in the treatment of RA and ulcerative colitis [4]. However, the efficacy and safety of TOF in patients with refractory TAK remain unclear. Here, we present the first case of successful TOF treatment in TAK refractory to glucocorticoids, immunosuppressants, TNF-α blockers and tocilizumab (TCZ), a humanized anti-IL-6 receptor (IL-6R) monoclonal antibody. Additionally, our case suggests that TOF in combination with MTX could be effective in TAK patients for tapering glucocorticoids and that the serum concentration of IL-6 might be a useful biological marker to assess the disease activity.

A 26-year-old man, diagnosed with TAK at the age of 24 years and treated with prednisolone (16 mg/day) in combination with infliximab (400 mg/8 weeks), ciclosporin (200 mg/day) and AZA (50 mg/day), was admitted to our hospital owing to a relapse of the disease. He was treated with prednisolone (30 mg/day) in combination with TCZ (162 mg/week), followed by oral CYC (100 mg/day) for the induction of remission. However, neither TCZ nor CYC was effective in tapering prednisolone to <25 mg/day, owing to aggravation of his chest pain. A CT scan revealed the worsening of arterial wall thickness of both the left common carotid artery and the descending aorta, with pleural effusion (Fig. 1A). In view of the inflammatory phenotype caused by multiple cytokines through cytokine receptors and their downstream Janus kinase/STAT signalling pathways, we treated the patient with 10 mg/day of TOF in combination with 50 mg/day of prednisolone based on approval from the Okayama University Hospital Ethics Committee. His symptoms improved after dissipation of the thickened aorta and pericardial pleural effusion (Fig. 1B), despite development of an infection twice (bacteraemia of unknown origin and atheroma infection; Fig. 1C). Notably, MTX in combination with TOF was effective against chest pain and the elevated serum CRP concentrations after tapering of prednisolone to 20 mg/day (Fig. 1C), demonstrating the additive effects of MTX and TOF in the maintenance of remission in TAK. After commencement of MTX, prednisolone was tapered further, from 20 to 15 mg/day, without relapse (Fig. 1C). Lastly, we performed a multiplex measurement of serum inflammatory cytokines, and observed high concentrations of IL-6, TNF-α and IL-18 during high disease activity. After the commencement of treatment, only the IL-6 concentration was decreased, suggesting a possible role of IL-6 as a biological marker of TAK (Fig. 1C).

**Key message**

- Tofacitinib in combination with MTX is a possible treatment strategy in refractory Takayasu arteritis.

**Figure:**
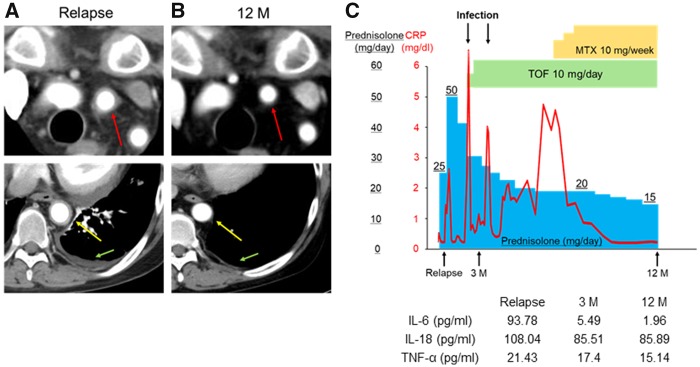
Fig. 1 CT scan images, clinical course and serum concentrations of cytokines in the present case (**A** and **B**) The CT images show the left common carotid artery (red arrow), the descending aorta (yellow arrow) and pleural effusion (green arrow) before (A; Relapse) and 12 months after (B; 12 M) the commencement of tofacitinib (TOF). (A) Prednisolone (25 mg/day) + CYC (100 mg/day). (B) Prednisolone (15 mg/day) + TOF (10 mg/day) + MTX (10 mg/week). (**C**) Clinical course of the patient, showing serum concentrations of CRP and inflammatory cytokines before and during 12 months of prednisolone and TOF treatment.

A recent randomized, placebo-controlled, double-blind, parallel-group, comparative study demonstrated that TCZ is effective in patients with refractory TAK, leading to clinical application [5]. However, 61% of TAK patients treated with TCZ relapse during the tapering of glucocorticoids [5], indicating that another therapeutic strategy needs to be established. Another recent study in mice suggested the clinical effectiveness of TOF in large vessel vasculitis [6]. Given that TOF is approved only for treatment of RA and ulcerative colitis in Japan, the off-label use of TOF in the present case was approved by the Okayama University Hospital Ethics Committee and supported financially by the Okayama University Hospital. Our case indicates that TOF in combination with MTX is a possible treatment strategy in patients with refractory TAK, and the serum concentration of IL-6 might be a useful biological marker to assess the disease activity.

In conclusion, our report provides new clinical insight into the role of TOF for refractory TAK and expands the concept that TOF in combination with MTX might be effective in other autoinflammatory diseases caused by multiple cytokines.

*Funding*: This work was not supported by any agency grants except for the cost of tofacitinib, which was supported by the Okayama University Hospital.

*Disclosure statement*: The authors have declared no conflicts of interest.
